# Two-Step Tuberculin Skin Testing in School-Going Adolescents with Initial 0-4 Millimeter Responses in a High Tuberculosis Prevalence Setting in South India

**DOI:** 10.1371/journal.pone.0071470

**Published:** 2013-09-06

**Authors:** Maitreyi Murthy, Sumithra Selvam, Nelson Jesuraj, Sean Bennett, Mark Doherty, Harleen M. S. Grewal, Mario Vaz, and the TB Trials Study Group

**Affiliations:** 1 Department of Clinical Science, Infection, Faculty of Medicine and Dentistry, University of Bergen; 2 Department of Microbiology, Haukeland University Hospital, Bergen, Norway; 3 St. John’s Research Institute, Bangalore, Karnataka, India; 4 Division of Epidemiology, Biostatistics and Population Health, St. John’s Research Institute, Bangalore, Karnataka, India; 5 Infectious Disease Epidemiology, St. John’s Research Institute, Bangalore, Karnataka, India; 6 Clinical Development, Aeras Global TB Vaccine Foundation, Rockville, Maryland, United States of America; 7 Department of Infectious Disease Immunology, GlaxoSmithKline, Copenhagen, Denmark; 8 Department of Clinical Science, Infection, Faculty of Medicine and Dentistry, University of Bergen and Department of Microbiology, Haukeland University Hospital, Bergen, Norway; 9 Health and Humanities, St. John’s Medical College and St. John’s Research Institute, Bangalore, Karnataka, India; Public Health Agency of Barcelona, Spain

## Abstract

**Background:**

The utility of two-step tuberculin skin testing among adolescents in high tuberculosis prevalence settings is not well established.

**Objectives:**

To determine the proportion and determinants of a 0-4 mm response to an initial standard tuberculin skin test (TST) and evaluating 'boosting' with repeat testing.

**Methods:**

Adolescents between 11 and 18 years attending schools/colleges underwent a TST; those with a response of between 0–4 mm had a repeat TST 1-4 weeks later.

**Results:**

Initial TST was done for 6608/6643 participants; 1257 (19%) developed a 0-4 mm response to the initial TST. Younger age and under-nutrition were more likely to be associated with a 0-4 mm response, while the presence of BCG (Bacillus Calmette Guerin) scar and higher socio-economic class were less likely to be associated with a 0-4 mm response. On repeat testing boosting was seen in 13.2% (145/1098; ≥ 6 mm over the initial test) while 4.3% showed boosting using a more conservative cutoff of a repeat TST ≥ 10 mm with an increment of at least 6 mm (47/1098). History of exposure to a tuberculosis (TB) case was associated with enhanced response.

**Conclusion:**

The proportion of adolescents who demonstrated boosting on two-step TST testing in our study was relatively low. As a result repeat testing did not greatly alter the prevalence of TST positivity. However, the two-step TST helps identify individuals who can potentially boost their immune response to a second test, and thus, prevents them from being misclassified as those with newly acquired infection, or tuberculin converters. While two-step tuberculin skin testing may have a limited role in population- level TST surveys, it may be useful where serial tuberculin testing needs to be performed to distinguish those who show an enhanced response or boosters from those who indeed have a new infection, or converters.

## Introduction

Tuberculosis (TB) accounts for the second highest number of deaths related to an infectious disease, globally, after HIV [1]. The vast majority of these deaths (95%) occur in low and middle income countries [1]. The burden of TB is concentrated in 22 countries, among which India has the highest number of cases, accounting for nearly 21% of the worldwide burden in terms of new cases occurring each year [1].

About one-third of the world is estimated to have latent TB infection (infected with *Mycobacterium tuberculosis* (M.tb), but do not have TB disease) [1]. Between 5–10% of those with latent TB infection will develop active TB if not treated, the risk being highest in the first 2 years after infection [2].

The tuberculin skin test (TST) (of which the Mantoux technique is most popularly employed) and interferon-gamma release assays (IGRAs) are the most commonly used tools to diagnose infection with *Mycobacterium tuberculosis* (M.tb) [2]. Test results may be affected by malnutrition, immune-suppressed states especially HIV (Human immunodeficiency virus) infection, age, gender, or prior exposure to mycobacteria (BCG (Bacillus Calmette Guerin) vaccination, tuberculous (M.tb) or non-tuberculous mycobacteria (NTMs)) [3–6]. While IGRAs have the benefit of increased specificity [7–9], the TST continues to be employed throughout the world. In a meta-analysis of T-cell based assays (including TST and Quantiferon (QFT), a type of IGRA based assay) to detect latent TB infection (LTBI) that included studies from low/middle income and high income countries, Pai M et al. showed that the pooled sensitivity of the QFT was 78%, while the TST was 77% [10]. The specificity analysis however included data from high income countries only, and showed that the pooled specificity of the QFT for BCG vaccinated populations was 96%, while non-BCG vaccinated showed 99% [10]. A more recent meta-analyses by Trajman et al., to compare the TST and QFT in determining LTBI reconciled findings for the QFT, as per the former analysis; and also showed that the pooled specificity for TST for BCG vaccinated was only 59%, while non-vaccinated was 97% [11]. However, none of the specificity studies had data from countries where the BCG vaccine was given during infancy. This gives us scope to study two-step tuberculin testing in our setting, which is not routinely done; and in a population who are routinely BCG vaccinated at birth.

The TST may be repeated within a short interval (1-5 weeks), to determine if an initial positive response reflects true infection [12]. Interpretation of these results is complicated by prior mycobacterial exposure of the individual which may produce false-positive responses or boosting [12]. Boosting is defined as a positive TST response following two-step testing, in the absence of new infection in the intervening period [12]. False positivity may also be due to variations in the administration/reading of the test. In this study we assessed the proportion and determinants of 0-4 mm TST responses in adolescents in South India. We then assessed the proportion and determinants of boosting following a second TST administered 1-4 weeks after the first; boosting being defined using two criteria; an increase of 6 mm or greater over the initial TST as the sole criterion [12], given that the intra-individual variability in TST responses is 6 mm [12] and an absolute value of 10 mm or more, with an increment of 6 mm or greater over the initial test.

## Methods

### Ethics and regulatory considerations

The study was reviewed and approved by two Ethics Review Boards (St. John’s Research Institute, Bangalore, India and an Independent Ethics Committee (IEC) of Aeras, Maryland, USA). The protocol was also approved by the Ministry of Health Screening Committee, Government of India. The studies were conducted after meeting with state and local education and health authorities. All subjects were enrolled after obtaining written parental consent. In addition, written assent from the adolescents was also obtained and study enrollment occurred only for those individuals from whom both parental consent and written assent was obtained. The signed consent forms were archived and a copy of the information sheet and consent form was given to the parent for their reference. Parents who were illiterate provided a thumb impression in the presence of a witness.

Consent of study participants was undertaken by staff trained in ICH-GCP guidelines and who underwent specific professional development modules in research ethics and the process of obtaining informed consent as part of in-house training.

### Design and study population

This study is a sub-study nested within a larger prospective cohort study. The principal objective of the larger study was to determine the two-year incidence of TB in a school-going adolescent population, as part of the development of a field study site for future TB vaccines. This study population was chosen since incidence data on this age group; a potential population for testing a post-exposure TB vaccine were unavailable. The study was conducted in Palamaner Taluk, which is a sub-district administrative area located in Chittoor District of Andhra Pradesh, South India and having a population of about 5,00,000. It is largely a rural community with some small towns and where agriculture, dairy and poultry farming are among the major sources of livelihood in the community.

### Subjects

Adolescents aged 11 to less than 18 years attending schools and junior colleges in the Taluk were eligible to participate in the study. This age group was chosen anticipating that they would be the target group for a future phase 3, post-exposure TB vaccine trial, as some data have shown that the risk of TB disease following infection begins to rise from the age of 12 until 19 years.

Subjects who were likely to move out of the study area over the following two years were excluded.

### Socio-economic and Clinical evaluation

At baseline, the socio-economic profile (parental education, type and quality of housing) of each participant was noted. A detailed clinical history including a present/past diagnosis of TB and reported BCG immunization was recorded. Anthropometric assessment included height and weight measurements.

#### Tuberculin skin testing

Following blood collection, a tuberculin test with 2 TU (Span Diagnostics, India) was administered to all participants by trained staff nurses. The TST was administered on the left forearm on the ventral surface, using the intradermal or Mantoux technique, until a wheal of 6-10 mm was visible at the injected area. Participants were instructed on care of the tested region, and informed about reading of the test reaction 2- 4 days afterwards.

The test was read using the ball-pen technique by either field staff or staff nurses who were trained in administration/reading of the tuberculin reaction. The staff were periodically retrained and their TST reading skills were randomly verified by the Field Supervisors and Medical Officers.

At follow up, those subjects, whose baseline TST reading measured 0-4 mm, received another TST within the following 1-4 weeks, in order to identify potential boosters to the second test. One hundred and fifty nine subjects were unavailable during follow-up for the following reasons; 9 participants reported sick, 31 refused the second test, 4 refused the second test due to Board Exams at school, 89 were not available for the reading of the second test and 26 were not available during the prescribed window period of 4 weeks following the first TST. While these subjects were excluded from the analysis, they continued to be evaluated as part of the larger cohort study as per protocol.

### Defining enhanced response following the two-step TST

Two cut-off measurements were used to define an increase in response following the repeat TST:

(i) An increase of 6 mm or greater over the initial TST as the sole criterion[12]
(ii) An absolute value of 10 mm or more, with an increment of 6 mm or greater over the initial test[12]

It has been shown that when the TST is repeated, chance variation is about 3 mm, or a value of 6 mm or more is considered a true biological response [12]. A cut-off of 10 mm is used by some, and by including an increment of 6 mm or more, we are thereby increasing the specificity of the reading obtained.

### Data management and analysis

Data were collected using standardized questionnaires. Double data entry was done on customized data acquisition software. SPSS version 18.0, SPSS Inc., Chicago, Illinois was used for analysis. BMI for age was computed using WHO – ANTHRO software (version 3.2.2), and for deriving BMI for age z scores to assess nutritional status. The data presented in this software is a pooled sample from 6 countries namely Brazil, Ghana, India, Norway, Oman and the USA. It consists of the WHO’s National Centre for Health Statistics (NCHS) data of 1977 that included growth of children and adolescents above 5 years of age ; that was merged with the records of the 18-71 year-olds of the WHO standards sample, resounding the fact that children grow similarly when their health and care needs are met.

Descriptive statistics are reported as numbers and percentages. The Chi-square or Fisher’s Exact test, as appropriate were used to test the association between the categorical variables (gender, age, socio-economic status, BCG status, BMI for age, and exposure to TB), in relation to an enhanced response to the second step TST, as well as for the association with a 0-4 mm response. Multivariate logistic regression was performed on the variables that were significant in the univariate analysis, to assess the factors associated with a 0-4 mm TST response, adjusting for socio-demographic and clinical variables. Adjusted odds ratios with 95% confidence intervals were reported. Level of significance was set at 5%.

## Results

### Population characteristics

**Figure 1:**
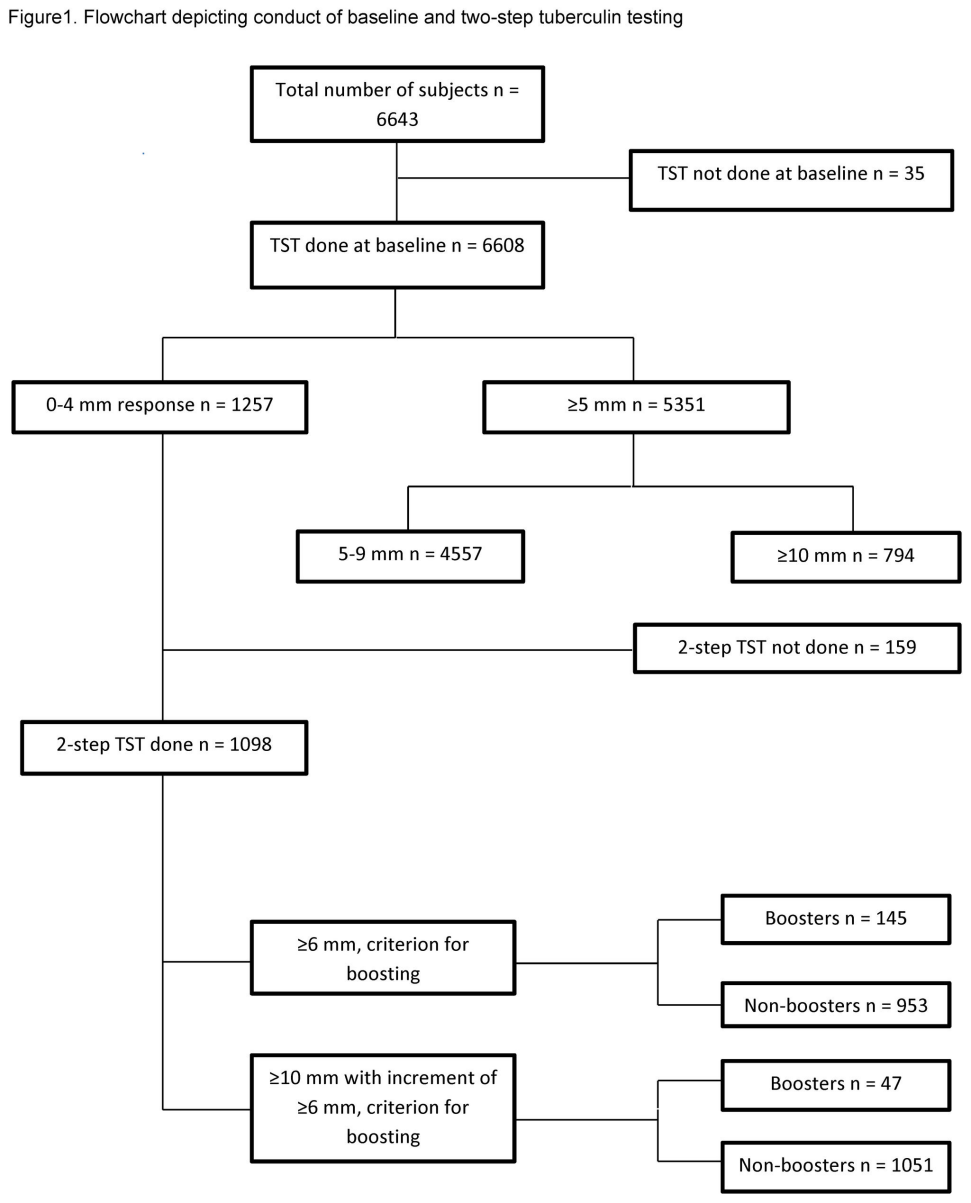
Flowchart depicting conduct of baseline and two-step tuberculin testing.

Of the total 12,388 eligible participants, 6643 adolescents were enrolled (response rate 53.6%). The male/ female ratio was 1.1:1 (51.5% vs. 48.2%). Figure 1 provides details of the participant enrollment and subsequent investigations. A history of BCG immunization was reported by 5674 (85.4%), 62.6% (4161) had visible scars and 2007 (30.2%) of the subjects were underweight. TST was administered to 6608/6643 (99.4%) participants at baseline. Of them, 1257 (19%) had a 0-4 mm response among which non-responders (0 mm) were 601 (9.1%), and those with intermediate responses (5-9 mm) were 4557/6608 (68.9%). Data were not obtained for the remaining 35 subjects, as they were not available for either administration/reading of the TST at baseline. Among those with a 0-4 mm response, 627/1257 (49.8%) were males.

### Characteristics of subjects with initial 0-4 mm responses and a subsequent booster response

Table 1 shows the association of 0-4 mm tuberculin responses with socio-demographic and clinical characteristics. Multivariate logistic regression analysis confirmed that younger participants {Adjusted Odds Ratio (AOR) = 1.61 (1.08-2.39)} were more likely to develop a 0-4 mm response as compared to the older subjects. Higher socio-economic status (determined by higher level of maternal education and living in houses whose walls were made of costlier materials such as brick) was less likely to be linked to a 0-4 mm response {AOR = 0.79 (0.68-0.92)}, as also was having visible BCG scars {AOR = 0.81 (0.71-0.91)}. Children who were underweight were more likely to respond with a 0-4 mm response to an initial TST {AOR = 1.16 (1.02-1.33)}.

**Table 1:** Association of 0-4 mm TST responses to an initial TST with socio-demographic and clinical characteristics.

Variables	Categories	0-4 mm (n = 1257)	≥5 mm (n = 5351)	p value	Unadjusted OR (95% CI)
Gender	Male	627 (49.9)	2787 (52.1)	0.16	0.92 (0.81-1.04)
	Female®	630 (50.1)	2564 (47.9)		
Age category	11-12	421 (33.5)	1357 (25.4)	<0.01	2.01 (1.34-3.04)
	13-14	659 (52.4)	2839 (53.1)		1.51 (1.01-2.26)
	15-16	146 (11.6)	954 (17.8)		0.99 (0.64-1.54)
	17-18®	31 (2.5)	201 (3.8)		1.00
Education of Mother*	Illiterate	644 (51.3)	2546 (47.6)	0.02	1.16 (1.02-1.31)
	Others®	612 (48.7)	2799 (52.4)		
Education of Father*	Illiterate	347 (27.8)	1377 (25.8)	0.16	1.10 (0.96-1.27)
	Others®	903 (72.2)	3953 (74.2)		
Religion	Hindu	1108 (88.1)	4743 (88.6)	0.62	0.95 (0.78-1.16)
	Others®	149 (11.9)	608 (11.4)		
Caste	Dalit/Harijan	212 (16.9)	1018 (19.0)	0.08	0.86 (0.73-1.02)
	Others®	1045 (83.1)	4333 (81.0)		
Type of walls*	Brick	951 (75.7)	4287 (80.1)	<0.01	0.77 (0.66-0.89)
	Others®	306 (24.3)	1062 (19.9)		
Type of fuel	Wood	1087 (86.5)	4578 (85.6)	0.40	1.08 (0.90-1.30)
	Others®	170 (13.5)	773 (14.4)		
BCG Immunization*	Yes	1061 (86.4)	4588 (87.3)	0.40	0.93 (0.77-1.11)
	No®	167 (13.6)	668 (12.7)		
BCG Scar	Present	718 (57.1)	3424 (64.0)	<0.01	0.75 (0.66-0.85)
	Absent®	539 (42.9)	1927 (36.0)		
BMI	Thin/Severe thin	422 (33.6)	1575 (29.4)	<0.01	1.21 (1.06-1.38)
	Normal or above ®	835 (66.4)	3776 (70.6)		
History of Contact	Yes	7 (0.6)	30 (0.6)	0.99	0.99 (0.40-2.37)
	No®	1250 (99.4)	5321 (99.4)		
History of Past TB	Yes	2 (0.2)	7 (0.1)	0.81	1.22†
	No®	1255 (99.8)	5344 (99.9)		
Symptoms of Cough‡	Yes	11 (0.9)	29 (0.5)	0.17	1.62 (0.76-3.39)
	No®	1246 (99.1)	5322 (99.5)		
Symptoms of Fever	Yes	4 (0.3)	14 (0.3)	0.73	1.22 (0.34-3.95)
	No®	1253 (99.7)	5337 (99.7)		
Symptoms of Weight loss	Yes	12 (0.9)	15 (0.3)	0.01	3.43 (1.50-7.76)
	No®	1245 (99.1)	5336 (99.7)		
Symptoms of Night sweats	Yes	5 (0.4)	4 (0.1)	<0.01	5.34 (1.25-23.61)
	No®	1252 (99.6)	5347 (99.9)		
Symptoms of Hemoptysis	Yes	3 (0.2)	4 (0.1)	0.11	3.20 (0.57-16.83)
	No®*	1254 (99.8)	5347 (99.9)		

Reported as numbers and percentages within parenthesis

^*^ Because data were not available on some individuals total numbers are lower than the number of subjects who participated in the study

BCG- Bacillus Calmette Guerin; BMI- Body Mass Index for Age; OR- Odds Ratio;

CI- Confidence Interval

® Reference group; ‡ Considered positive if either parent/subject answered ‘yes’

† Confidence limits not reported due to small numbers

Table 2 shows that 145/1098 (13.2%) developed enhanced responses to a repeat TST using an increment of ≥ 6 mm as the sole criterion. A history of contact, religion as well as type of fuel used for cooking (wood compared to kerosene or other types, the latter indicating higher socio-economic class) was associated with an enhanced response. After adjusting for other factors, the multivariate analysis showed that those with a TB contact history were more than 4 times likely to develop an enhanced TST response {AOR = 4.45 (0.98-21.15)}.

**Table 2:** Association of enhanced TST responses following the second tuberculin skin test (boosters; ≥ 6 millimeter increase) with socio-demographic and clinical characteristics.

Variables	Categories	Enhanced response	Others	p value	Unadjusted OR
		(≥6 mm)	(0-5 mm)		(95% CI)
		(n = 145)	(n = 953)		
Gender	Male	69 (47.5)	464 (48.7)	0.80	0.95 (0.67-1.35)
	Female®	76 (52.4)	489 (51.3)		
Age category	11-12	50 (34.5)	334 (35.0)	0.90	1.27 (0.27-8.23)
	13-14	76 (52.4)	508 (53.3)		1.27 (0.27-8.13)
	15-16	17 (11.7)	94 (9.9)		1.54 (0.30-10.59)
	17-18®	2 (1.4)	17 (1.8)		1.00
Education of mother*	Illiterate	74 (51.0)	477 (50.1)	0.84	0.96 (0.67-1.36)
	Others®	71 (49.0)	475 (49.9)		
Education of father*	Illiterate	45 (31.3)	254 (26.8)	0.27	1.24 (0.84-1.81)
	Others®	99 (68.7)	693 (73.2)		
Religion	Hindu	119 (82.1)	844 (88.6)	0.03	0.59 (0.37-0.95)
	Others®	26 (17.9)	109 (11.4)		
Caste	Dalit/Harijan	22 (15.2)	153 (16.1)	0.79	0.94 (0.58-1.52)
	Others®	123 (84.8)	800 (83.9)		
Type of walls	Brick	110 (75.9)	720 (75.6)	0.94	1.01 (0.67-1.53)
	Others®	35 (24.1)	233 (24.4)		
Type of fuel	Wood	112 (77.2)	833 (87.4)	<0.01	0.48 (0.32-0.75)
	Others®	33 (22.8)	120 (12.6)		
BCG Immunization*	Yes	130 (91.5)	807 (86.7)	0.10	1.66 (0.89-3.09)
	No®	12 (8.5)	124 (13.3)		
BCG Scar	Present	89 (61.4)	540 (56.7)	0.28	1.21 (0.85-1.74)
	Absent®	56 (38.6)	413 (43.3)		
BMI	Thin/Severe thin	46 (31.7)	322 (33.8)	0.62	0.91 (0.62-1.32)
	Normal or Overweight®	99 (68.3)	631 (66.2)		
History of Contact	Yes	3 (2.1)	4 (0.4)	0.02	5.01 (1.11-22.6)
	No®	142 (97.9)	949 (99.6)		
History of Past TB	Yes	0(0)	2 (0.2)	0.58	-
	No®	145 (100.0)	951 (99.8)		
Symptoms of Cough	Yes	1 (0.7)	9 (0.9)	0.76	0.73 (0.09-5.79)
	No®	144 (99.3)	449 (99.1)		
Symptoms of Fever	Yes	1 (0.7)	3 (0.3)	0.48	2.19†
	No®	144 (99.3)	950 (99.7)		
Symptoms of Weight loss	Yes	1 (0.7)	11 (1.2)	0.61	0.59 (0.07-4.64)
	No®	144 (99.3)	942 (98.8)		

Reported as numbers and percentages within parenthesis

Multivariate analysis: adjusted for age, gender, type of fuel used (surrogate of socio-economic status), BCG immunization, contact (surrogate of exposure to TB), BMI for age (surrogate of nutritional status); details in text.

^*^ Because data were not available on some individuals total numbers are lower than the number of subjects who participated in the study

BCG- Bacillus Calmette Guerin; BMI- Body Mass Index for Age; OR- Odds Ratio

CI- Confidence Interval

® Reference group; ‡ Considered positive if either parent/subject answered ‘yes’

† Confidence limits not reported due to small numbers

**Table 3:** Association of boosting following the second tuberculin skin test (Boosters; ≥ 10mm with an increment of ≥ 6 mm following a two-step TST) with socio-demographic and clinical characteristics.

Variables	Categories	Boosters (≥ 10 mm with an increment of ≥ 6 mm) (n = 47)	Non-boosters (n = 1051)	p value	Unadjusted OR (95% CI)
Gender	Male	18 (38.3)	515 (49.0)	0.15	0.65 (0.34-1.22)
	Female®	29 (61.7)	536 (51.0)		
Age category	11-12	16 (34.0)	368 (35.0)	0.93	0.78 (0.10-16.67)
	13-14	24 (51.1)	560 (53.3)		0.77 (0.10-16.13)
	15-16	6 (12.8)	105 (10.0)		1.03 (0.11-24.02)
	17-18®	1 (2.1)	18 (1.7)		1.00
Education of mother*	Illiterate	23 (48.9)	528 (50.3)	0.80	0.95 (0.51-1.76)
	Others®	24 (51.1)	522 (49.7)		
Education of father*	Illiterate	17 (36.2)	282 (27.0)	0.17	1.53 (0.80-2.93)
	Others®	30 (63.8)	762 (73.0)		
Religion	Hindu	38 (80.9)	925 (88.0)	0.14	0.58 (0.26-1.31)
	Others®	9 (19.1)	126 (12.0)		
Caste	Dalit/Harijan	6 (12.8)	169 (16.1)	0.54	0.76 (0.29-1.91)
	Others®	41 (87.2)	882 (83.9)		
Type of walls	Brick	39 (82.9)	791 (75.3)	0.22	1.60 (0.71-3.76)
	Others®	8 (17.1)	260 (24.7)		
Type of fuel	Wood	38 (80.9)	907 (86.3)	0.29	0.67 (0.30-1.52)
	Others®	9 (19.1)	144 (13.7)		
BCG Immunization*	Yes	39 (86.7)	898 (87.4)	0.89	0.94 (0.37-2.52)
	No®	6 (13.3)	130 (12.6)		
BCG Scar	Present	30 (63.8)	599 (57.0)	0.35	1.33 (0.70-2.55)
	Absent®	17 (36.2)	452 (43.0)		
BMI	Thin/Severe thin	17 (36.2)	351 (33.4)	0.69	1.13 (0.59-2.15)
	Normal or Overweight®	30 (63.8)	700 (66.6)		
History of Contact	Yes	1 (2.2)	6 (0.6)	0.19	3.79†
	No®	46 (97.8)	1045 (99.4)		
History of Past TB	Yes	0(0)	2 (0.2)	0.76	-
	No®	47 (100.0)	1049 (99.8)		
Symptoms of Cough	Yes	1 (2.2)	9 (0.9)	0.37	2.52†
	No®	46 (97.8)	1042 (99.1)		
Symptoms of Fever	Yes	1 (2.2)	3 (0.3)	0.04	7.59†
	No®	46 (97.8)	1048 (99.7)		
Symptoms of Weight loss	Yes	1 (2.2)	11 (1.1)	0.49	2.06†
	No®	46 (97.8)	1040 (98.9)		

Reported as numbers and percentages within parenthesis

Multivariate analysis: adjusted for age, gender, type of walls of house (surrogate of socio-economic status), BCG immunization and BCG scar, contact (surrogate of exposure to TB), BMI for age (surrogate of nutritional status); details in text.

^*^ >Because data were not available on some individuals total numbers are lower than the number of subjects who participated in the study

BCG- Bacillus Calmette Guerin; BMI- Body Mass Index for Age; OR- Odds Ratio;

CI- Confidence Interval

® Reference group; ‡ considered positive if either parent/subject answered ‘yes’

† Confidence limits not reported due to small numbers

Table 3 shows that 47/1098 (4.3%) of the participants developed enhanced responses of ≥ 10 mm with an increment of 6 mm or more following a repeat TST. A history of contact, though not statistically significant at the univariate level, was significantly associated with an enhanced TST response after adjusting for clinical and socio-demographic characteristics in the multivariate logistic regression model {AOR = 7.59 (1.46-39.5)}.

Adjusted Odds ratios were calculated for the variables significant in the univariate analysis. It was seen that older individuals were more likely to show boosting, even within the relatively constrained age-ranges in our study {AOR = 1.11 (0.24-5.1), 1.09 (0.24-4.97) and 1.43 (0.29-6.99)} and {AOR = 0.73 (0.90-5.94), 0.72 (0.91-5.71) and 1.03 (0.12-9.26)} for the age groups 11-12, 13-14 and 15-16 respectively; for the first and second definitions of boosting. Having a BCG scar had no effect on the booster response.

Boosting was seen in 4.3% to 13.2% of subjects who had initial 0-4 mm responses. TST positivity increased from 12.7%-14.2%, from a baseline value of 12 .0%, while including those who boosted their TST -47 and 145 subjects respectively, to the 794 who were already positive (≥ 10 mm) at baseline.

## Discussion

The two-step TST method, although not commonly practiced in our country, is employed routinely in low TB burden settings among populations who will be subject to periodic retesting, namely health-care workers and others with high risk of on-going exposure, such as prison inmates and day-care workers. Such an approach reduces the chance that a second positive TST will be interpreted as a new conversion, when it in fact, represents a boosted response [13]. The prevalence of boosting varies widely; depending on the prevalence of TB in the population and/or background infection with NTMs, as well as BCG vaccination coverage in that population [14]. In our study, we recorded a prevalence of boosting between 4.3% and 13.2% using the two different cut-off criteria. We observed that a history of contact with a TB case was linked to boosting in our study sample.

Increasing age is associated with the booster phenomenon [14,15]. There was a trend towards increasing booster responses within the relatively constrained age-ranges in our study; as per both definitions of boosting. In contrast, a study among children aged 6 months to 14 years of age showed that the booster response was highest among those aged 6 months to 6 years, who also had BCG scars [16]. The cross-reaction of BCG with TST may explain this finding. There was no association with BCG scar and boosting in the older subjects [16], and this may be because the effect of BCG on immune response wanes with time. Our study, conducted among adolescents within a relatively small age range (11-18 years) demonstrated an increased likelihood of boosting with increasing age- especially the 15-16 year old group. Prior exposure to mycobacteria, including other determinants namely socio-cultural factors, employment and migration seen during this age period may also account for variations in prevalence of boosting.

BCG shares common antigens with those contained in tuberculin and therefore results in cross-reactions when a BCG vaccinated person is tuberculin tested, though factors linked to BCG vaccination itself, which include the type of vaccine, the number of vaccinations, the dosage administered, the age at vaccination, route of vaccination and the time elapsed since vaccination may also affect the extent of cross-reaction with TST [5,15,17–21]. Studies by Silva VMC [22] et al. and Srour-Fihmi S et al. [23] in age groups ranging from 20 years to over 60 years have shown that being BCG vaccinated had no effect on the booster response. Studies also show that the effect of BCG is insignificant when TST reactions are ≥ 10mm [5,6,18,24–26].

Most of our study subjects have received BCG vaccination during infancy (as per national immunization guidelines), and re-vaccination is not routine practice in India. The results from our study on BCG vaccinated subjects are consistent with the literature, where it is generally reported that routine BCG vaccination during infancy has a minimal effect on the tuberculin response in adulthood [14,21,27].

Boosting is commonly seen in populations in tropical climate conditions, where background sensitization with NTMs is high [28,29]. The prevalence of NTMs in India varies from 0.5–8.6% [30], although community based studies are few and there is likely to be heterogeneity across geographical regions and communities. In the present study, only 3 (0.1%) of those with an enhanced response following repeat testing had documented sensitization with NTMs during the study period based on sputum culture, although this does not preclude earlier sensitization (data not shown). It is imperative to mention here that very little is known about NTMs and what impact they may have on boosting, given that the references (28,29) are quite dated. As a matter of fact, WHO has more or less asked that data from those surveys in the late 1950’s to early 1960’s be ignored given the time, and methodology used relative to this period.

A history of exposure to M.tb has also been associated with the booster phenomenon. None of the subjects had reported exposure to an infectious case during the study period (we maintained record of this event during each follow-up visit, as well as the period in between the follow-ups). Moreover, we repeated the test within 1-4 weeks, thereby eliminating the possibility that an enhanced response to the repeat test be misclassified as a conversion, when in fact it was merely a boosted response.

Of those with a history of contact, only one developed a boosted response, when the criterion for defining a booster response as ≥ 10 mm, with an increment of ≥ 6 mm was used, making it difficult to conclude that remote infection is associated with boosting. However, when only an increment of ≥ 6 mm was used, 3 participants who demonstrated boosting had a history of contact {AOR = 5.86 (1.15-29.72), p value 0.02}. This may imply that with a smaller cut-off (increasing sensitivity- albeit at the cost of decreasing specificity), more boosters may be identified. Annual tuberculin testing is not warranted in casual contacts and low-risk groups as the chances of being truly infected with M.tb are minimum at best, but the two-step test might be useful to separate individuals who show boosted responses from those at higher risk (household contacts, HIV infected, Health care workers) who are more likely to develop true conversions due to recent M.tb exposure.

## Conclusion

The proportion of adolescents with a 0-4 mm response who demonstrated boosting on two-step TST testing in our study was relatively low. TST positivity did not seem to increase meaningfully when those already positive (≥10 mm) at baseline were also included. This does not greatly alter the prevalence of TST positivity in a high TB prevalence setting. However, the two-step TST helps identify individuals who can potentially boost their immune response to a second test, and thus, prevents them from being misclassified as those with newly acquired infection, or tuberculin converters.

Thus, while two-step tuberculin skin testing may have a limited role in population- level TST surveys, it may be useful in settings where serial tuberculin testing will be performed to distinguish those who show an enhanced response or boosters from those who indeed have a new infection, or converters.

## References

[1] World Health Organization (2012) uberculosis. Available: http://www.who.int/mediacentre/factsheets/fs104/en/index.html. Accessed 11 June 2012.

[2] CDC (2012) asic TB Facts. Available: http://www.cdc.gov/tb/topic/basics/risk.htm. Accessed 11 June 2012.

[3] Gallant CJ, Cobat A, Simkin L, Black GF, Stanley K et al. (2010) Impact of age and sex on mycobacterial immunity in an area of high tuberculosis incidence. Int J Tuberc Lung Dis 14: 952-959. PubMed: 20626938. 20626938

[4] Shanaube K, Hargreaves J, Fielding K, Schaap A, Lawrence K-A et al. (2011) Risk factors associated with positive Quantiferon –TB Gold In –Tube and tuberculin skin tests results in Zambia and South Africa. PLOS ONE 6: e18206. doi:10.1371/journal.pone.0018206. PubMed: 21483746. 21483746 10.1371/journal.pone.0018206 PMC3070723

[5] Saito M, Bautista CT, Gilman RH, Bowering A, Levy MZ et al. (2004) The value of counting BCG scars for interpreting tuberculin skin tests in a tuberculosis hyperendemic shanty-town, Peru. Int J Tuberc Lung Dis 8: 842-847. PubMed: 15260275. 15260275 PMC2912512

[6] Santiago EM, Lawson E, Gillenwater K, Kalang Si Lescano AG et al. (2003) A prospective study of Bacillus Calmette –Guerin scar formation and tuberculin skin test reactivity in infants in Lima, Peru. Pediatrics 112: 298-302. doi:10.1542/peds.112.4.e298. 10.1542/peds.112.4.e298 14523215

[7] Menzies D, Pai M, Comstock G (2007) Meta-analysis: new tests for the diagnosis of latent tuberculosis infection: areas of uncertainty and recommendations for research. Ann Intern Med 146: 340-354. doi:10.7326/0003-4819-146-5-200703060-00006. PubMed: 17339619. 17339619 10.7326/0003-4819-146-5-200703060-00006

[8] Casas I, Latorre I, Esteve M, Ruiz-Manzano J, Rodriguez D et al. (2009) Evaluation of interferon-gamma release assays in the diagnosis of recent tuberculosis infection in health care workers. PLOS ONE 4: e6686. doi:10.1371/journal.pone.0006686. PubMed: 19701460. 19701460 10.1371/journal.pone.0006686 PMC2726945

[9] González-Salazar F, Vargas-Villarreal J, Garcialuna-Martínez FJ, Rivera G, Moreno-Treviño MG et al. (2011) Snapshot of Quantiferon TB gold testing in Northern Mexico. Tuberculosis 91: S34-S37. doi:10.1016/j.tube.2011.10.007. PubMed: 22099419. 22099419 10.1016/j.tube.2011.10.007

[10] Pai M, Zwerling A, Menzies D (2008) Systematic Review: T-Cell–based Assays for the Diagnosis of Latent Tuberculosis Infection: An Update. Ann Intern Med 149: 177-184. doi:10.7326/0003-4819-149-3-200808050-00241. PubMed: 18593687. 18593687 10.7326/0003-4819-149-3-200808050-00241 PMC2951987

[11] Trajman A, Steffen RE, Menzies D (2013) Interferon-Gamma Release Assays versus Tuberculin Skin Testing for the Diagnosis of Latent Tuberculosis Infection: An Overview of the Evidence. Pulm Med, 2013: 2013:1-11 PubMed: 23476763. 10.1155/2013/601737 PMC3582085 23476763

[12] Menzies D (1999) Interpretation of repeated tuberculin tests. AJRCCM 159: 15-21. 10.1164/ajrccm.159.1.9801120 9872812

[13] CDC (2012) Tuberculosis: Tuberculin skin testing. Available: http://www.cdc.gov/tb/publications/factsheets/testing/skintesting.htm. Accessed 12 June 2012.

[14] Abdulrahman MA (2004) Booster effect of two-step tuberculin testing among hospital employees from areas with a high prevalence of tuberculosis. Infect Control Hosp Epidemiol 25: 1117-1119. doi:10.1086/502355. PubMed: 15636304. 15636304 10.1086/502355

[15] Menzies R, Vissandjee B, Rocher I, St Germain Y (1994) The booster effect in two-step testing among young adults in Montreal. Ann Intern Med 120: 190-198. doi:10.7326/0003-4819-120-3-199402010-00003. PubMed: 8273982. 8273982 10.7326/0003-4819-120-3-199402010-00003

[16] Friedland IR (1990) The booster effect with repeat tuberculin testing in children and its relationship to BCG vaccination. S Afr Med J 77: 387-389. PubMed: 2330523. 2330523

[17] Huebner RE, Schein MF, Hall CA, Barnes SA (1994) Delayed-type hypersensitivity anergy in human immunodeficiency virus-infected persons screened for infection with Mycobacterium tuberculosis. Clin Infect Dis 19: 26-32. doi:10.1093/clinids/19.1.26. PubMed: 7948554. 7948554 10.1093/clinids/19.1.26

[18] Sakha K, Behbahan AG (2008) Immunogenicity of neonatal BCG vaccination in children entering primary school. Pak J Biol Sci 11: 930-933. doi:10.3923/pjbs.2008.930.933. PubMed: 18814659. 18814659 10.3923/pjbs.2008.930.933

[19] Roth A, Sodemann M, Jensen H, Poulsen A, Gustafson P et al. (2005) Vaccination technique, PPD reaction and BCG scarring in a cohort of children born in Guinea-Bissau 2000-2002. Vaccine 23: 3991-3998. doi:10.1016/j.vaccine.2004.10.022. PubMed: 15899539. 15899539 10.1016/j.vaccine.2004.10.022

[20] Horowitz HW, Luciano BB, Kadel JR, Wormser GP (1995) Tuberculin skin test conversion in hospital employees vaccinated with bacilli Calmette- Guerin: recent Mycobacterium tuberculosis infection or booster effect? Am J Infect Control 23: 181-187. doi:10.1016/0196-6553(95)90039-X. PubMed: 7677263. 7677263 10.1016/0196-6553(95)90039-x

[21] Teixeira EG, Kritski A, Ruffino-Netto A, Steffen R, Lapa E et al Silva JR. (2008) Two-step tuberculin skin test and booster phenomenon prevalence among Brazilian medical students. Int J Tuberc Lung Dis 12: 1407-1413. PubMed: 19017450. 19017450

[22] Silva VMC, Cunha AJLA, Oliveira JR, Figueira MM, Nunes ZB et al. (2000) Medical students at risk of nosocomial transmission of Mycobacterium tuberculosis. Int J Tuberc Lung Dis 4: 420-426. PubMed: 10815735. 10815735

[23] Srour-Fihmi S, Weiler-Ravell D, Kitzes R, Chemtob D (2000) Routine two-step testing for tuberculosis in the staff of a geriatric hospital in Israel: booster and conversion rates. J Hosp Infect 46: 141-146. doi:10.1053/jhin.2000.0787. PubMed: 11049708. 11049708 10.1053/jhin.2000.0787

[24] Garcia-Sancho F, Ma C, Garcia-Garcia L, Jimenez-Corona Ma E, Palacios- Martinez M et al. (2006) Is tuberculin skin testing useful to diagnose latent tuberculosis in BCG-vaccinated children. International Journal of Epidemiology 35: 1447-1454. 17008360 10.1093/ije/dyl213

[25] Agrawal S (1998) Interpretation of Mantoux test. Indian Pediatr 35: 582-584. PubMed: 10216667. 10216667

[26] Chan PC, Chang LY, Wu Y-C, Lu C-Yi, Kuo H-S et al. (2008) Age specific cut-offs for the tuberculin skin test to detect latent tuberculosis in BCG-vaccinated children. Int J Tuberc Lung Dis 12: 1401-1406. PubMed: 19017449. 19017449

[27] Hizel K, Maral I, Karakus R, Aktas F (2004) The influence of BCG immunisation on tuberculin reactivity and booster effect in adults in a country with a high prevalence of tuberculosis. Clin Microbiol Infect 10: 980–983. doi:10.1111/j.1469-0691.2004.00970.x. PubMed: 15522000. 15522000 10.1111/j.1469-0691.2004.00970.x

[28] Organization WH (1955) Further studies of geographic variation in naturally acquired tuberculin sensitivity. Bull Wld Hlth Org 22: 63-83. PMC2542330 14351968

[29] Narain R, Anantharaman DS, Diwakara AM (1974) Prevalence of nonspecific tuberculin sensitivity in certain parts of India. Bull Wld Hlth Org 51: 273-278. PubMed: 4218969. PMC2366290 4218969

[30] Jani MN, Rodrigues CS, Mehta AP (2011) The neglected and often ignored: Nontuberculous mycobacteria. J Global Infect Dis 3: 94. doi:10.4103/0974-777X.77305. PubMed: 21572618. 10.4103/0974-777X.77305 PMC3068589 21572618

